# NKG2D is a Key Receptor for Recognition of Bladder Cancer Cells by IL-2-Activated NK Cells and BCG Promotes NK Cell Activation

**DOI:** 10.3389/fimmu.2015.00284

**Published:** 2015-06-08

**Authors:** Eva María García-Cuesta, Sheila López-Cobo, Mario Álvarez-Maestro, Gloria Esteso, Gema Romera-Cárdenas, Mercedes Rey, Robin L. Cassady-Cain, Ana Linares, Alejandro Valés-Gómez, Hugh Thomson Reyburn, Luis Martínez-Piñeiro, Mar Valés-Gómez

**Affiliations:** ^1^Department of Immunology and Oncology, National Centre for Biotechnology (CNB-CSIC), Madrid, Spain; ^2^Urology Unit, Infanta Sofía Hospital, Madrid, Spain; ^3^Department of Pathology, University of Cambridge, Cambridge, UK; ^4^Jobssy, S.L., Madrid, Spain

**Keywords:** natural killer cells, NKG2D, natural cytotoxicity receptors, *Mycobacterium bovis*, bladder cancer

## Abstract

Intravesical instillation of bacillus Calmette–Guérin (BCG) is used to treat superficial bladder cancer, either papillary tumors (after transurethral resection) or high-grade flat carcinomas (carcinoma *in situ*), reducing recurrence in about 70% of patients. Initially, BCG was proposed to work through an inflammatory response, mediated by phagocytic uptake of mycobacterial antigens and cytokine release. More recently, other immune effectors such as monocytes, natural killer (NK), and NKT cells have been suggested to play a role in this immune response. Here, we provide a comprehensive study of multiple bladder cancer cell lines as putative targets for immune cells and evaluated their recognition by NK cells in the presence and absence of BCG. We describe that different bladder cancer cells can express multiple activating and inhibitory ligands for NK cells. Recognition of bladder cancer cells depended mainly on NKG2D, with a contribution from NKp46. Surprisingly, exposure to BCG did not affect the immune phenotype of bladder cells nor increased NK cell recognition of purified IL-2-activated cell lines. However, NK cells were activated efficiently when BCG was included in mixed lymphocyte cultures, suggesting that NK activation after mycobacteria treatment requires the collaboration of various immune cells. We also analyzed the percentage of NK cells in peripheral blood of a cohort of bladder cancer patients treated with BCG. The total numbers of NK cells did not vary during treatment, indicating that a more detailed study of NK cell activation in the tumor site will be required to evaluate the response in each patient.

## Introduction

Intravesical instillation with Bacillus Calmette–Guérin (BCG), a live attenuated form of *Mycobacterium bovis*, is a first choice therapy for high-grade superficial bladder cancer, such as carcinoma *in situ* (CIS), and it is also used as an adjuvant, following transurethral resection (TUR), in papillary non-muscle invasive bladder cancer (NMIBC) (1). Due to the efficiency of this therapy, multiple studies on its mechanism of action have been published in the last decade [reviewed in Ref. (2)]; however, a full understanding of how BCG therapy works has still not been achieved (3). A detailed knowledge of the molecular basis of this therapy would be valuable since exposure to the bacteria can also produce undesired adverse effects and the ability to identify non-responder patients at an early stage would allow clinicians to switch to alternative therapies earlier in the disease. A considerable body of experimental evidence indicates that natural killer (NK) and NKT cells play an important role on the anti-tumor responses induced by BCG in the treatment of superficial bladder carcinoma (4–9). For this reason, it is important to study in detail the characteristics of the immune response mediated by these cells.

Natural killer cells usually represent 5–15% of peripheral blood lymphocytes and respond to their targets without prior antigen sensitization; in particular, their activity is important against virus-infected cells and tumor cells [for review, see Ref. (10)]. NK cell cytotoxicity is exerted by release of lytic granules toward the target cell after formation of a cytotoxic immunological synapse, but, instead of depending on a single receptor, like the TCR in T cells, NK cells require the integration of signals produced by a large number of activating and inhibitory receptors [for review, see Ref. (11, 12)]. In this context, while recognition of MHC-I can be a very strong inhibitory signal, activation mediated by certain activating receptors and cytokines can override the recognition of MHC. The presence of different receptors at the NK cell surface defines several NK subsets with different functional properties. Therefore, to understand how NK cells respond against bladder tumors, in the context of BCG, it is necessary to dissect the contribution of different receptors to NK cell recognition of these tumor cells.

Previous data show that NK cells could kill one urothelial tumor cell line, T24 (6, 7) and that effector cells could be recognizing NKG2D ligands on this cell line (9); however, the presence of these ligands in bladder cancer cells has not been previously explored. The purpose of the work presented here was to systematically evaluate the contribution of immune activating and inhibitory ligands to bladder cancer recognition by NK cells in the context of BCG immunotherapy. We report a comprehensive analysis of the immune phenotype of a panel of urothelial tumor cells and their differential ability to be lysed by primary NK cell lines from healthy donors. We identify NKG2D as a key receptor involved in the recognition of bladder cancer cells by activated NK cells, while NKp46 only contributes partially to the response against certain bladder cell lines. The exposure of purified NK cells to BCG does not affect NK function; however, activation of NK cells can be achieved by exposing PBMCs to BCG. Initial analysis of peripheral blood obtained from BCG-treated bladder cancer patients included in a pilot study show more differences in the percentage of NK cells among individuals than in response to treatment. We have also analyzed the amount of NKG2D receptor in the surface of different subpopulations of NK cells.

## Materials and Methods

### Reagents

#### Antibodies

Monoclonal antibodies specific for ULBP1, 2, 3, MICA, and MICB were purchased from R&D Systems (Abingdon, UK); ICAM-1/CD54 (Immunotech, Clone 84H10); Nectin 2/CD112 (Santa Cruz, Clone B-C12); CD155/PVR (Abcam, Clone D171); E-Cadherin (Immunotech, Clone 67A4); CD58/LFA-3 (Immunotech, Clone AICD58); CD106/VCAM-1 (Pharmingen, Clone 51-10C9); CD48 (Diaclone, Clone MEM102). MHC class I specific antibody [HP-1F7 (13)] and L31 anti-HLA-C antibody (14) were previously described. Conjugated antibodies for blood lymphocyte subpopulations were from Biolegend and Immunotools. Secondary antibodies, such as FITC- and PE-conjugated anti-mouse Ig, were purchased from DakoCytomation. Blocking antibodies, specific for anti-human NKG2D (clone 149810), NKp46/NCR1 (clone 195314), NKp30/NCR3 (clone 210845), and DNAM-I/CD226 (clone 102511), were purchased from R&D. KLRG1 antibody was kindly provided by Prof. H. Pircher (University Medical Center Freiburg).

Fusion proteins of NKp46 and NKp30 were prepared by exchanging the human Fc portion of the constructs previously described (15, 16) with a murine IgG1 Fc, to minimize possible Fc/Fc receptor interactions during flow cytometry of human cells. Stable transfectants secreting recombinant proteins were produced in HEK293FT cells (Life Technologies) maintained in DMEM 4.5 mg/l glucose medium supplemented with 10% FBS, l-Glutamine, non-essential amino acids, sodium pyruvate, and antibiotics (penicillin/streptomycin), and 1 μg/ml puromycin. NKG2D-Fc constructs, prepared by transient transfection in 293T cells, have been previously described (17).

Bacillus Calmette–Guérin Tice strain (from Merck Canada Inc.) was used for the majority of the experiments. For some experiments, the strains Connaught (from Sanofi Pasteur Limited) and Danish 1331 (kindly provided by Pfizer) were used. Aliquots of reconstituted BCG were prepared and stored at −20°C.

### Cell lines and peripheral blood subpopulations

The bladder cancer cell lines T24, UM-UC-3, J82, RT-112, RT4, and SW780 are available from the ATCC [cell lines were kindly provided by Dr. FX Real (CNIO, Madrid) and genotyped using the StemElite ID System (Promega) at the Genomics Service (IIB-CSIC)]. Cells were grown in EMEM medium (Lonza) supplemented with 10% FCS, 1 mM glutamine, 1 mM sodium pyruvate, 0.1 mM non-essential amino acids, 100 U/ml penicillin, and 100 U/ml streptomycin (Biowest).

Polyclonal NK cell lines were prepared from healthy volunteer buffy coats [(Regional Transfusion Centre, Madrid), approval from local ethical committees and informed consent from all participants were obtained; experiments have been conducted according to the principles expressed in the Declaration of Helsinki]. PBMCs were isolated by centrifugation on Ficoll-HyPaque. NK cells were isolated by negative selection using the MACS NK Cell Isolation Kit (Miltenyi Biotec) and cultured in the presence of irradiated feeder cells (autologous PBMCs, Daudi and RPMI-8866 B cell lines) in RPMI-1640 medium (Lonza) supplemented with 10% FBS, 10% male AB negative human serum (the first week, and later with 5% FBS, 5% Human Serum), 4 mM l-glutamine, 0.1 mM non-essential amino acids, 1 mM sodium pyruvate, 100 U/ml penicillin, 100 U/ml streptomycin, 10 mM Hepes, 50 μM β-mercaptoethanol (Biowest), and 50 IU/ml rhlL-2 (Peprotech). Greater than 95% pure NK cell cultures were re-stimulated weekly and the NK phenotype was monitored by flow cytometry. Functional experiments with NK cells were performed 4–5 days after IL-2 stimulation. Freshly isolated NK cells, after MACS purification, were maintained in culture, without rhIL-2, with or without BCG, up to 2 days, before use in experiments.

K562, Daudi, and RPMI-8866 cells were grown in RPMI-1640 medium (Lonza) supplemented with 10% FBS, 4 mM l-glutamine, 0.1 mM non-essential amino acids, 1 mM sodium pyruvate, 100 U/ml penicillin, 100 U/ml streptomycin, and 10 mM Hepes.

For experiments using mixed culture of lymphocytes, PBMCs were incubated for a week in the presence or absence of BCG in RPMI with 5% FBS, 5% Human Serum, 4 mM l-glutamine, 0.1 mM non-essential amino acids, 1 mM sodium pyruvate, 100 U/ml penicillin, 100 U/ml streptomycin, 10 mM Hepes, and 50 μM β-mercaptoethanol (Biowest).

### Flow cytometry

Cells were incubated with the appropriate primary antibodies or receptor-Fc fusion proteins, followed by either PE- or FITC-labeled F(ab′)2 fragments of goat anti-mouse Ig (Dako) or PE-labeled F(ab′)2 fragments of goat anti-human Fc (Immunotech), or directly with the conjugated antibodies. Samples were analyzed using BD FACSCalibur (Becton Dickinson), Gallios Flow Cytometer or Cytomics FC 500 (Beckman Coulter). Analysis of the experiments was performed using the Kaluza, Summit, or FlowJo softwares.

### Degranulation assays

Natural killer cells were co-cultured with target cells for 2 h at an E:T ratio of 1:2. Surface expression of LAMP-1 (CD107a) was analyzed by flow cytometry. In experiments where NK cell receptors were blocked, mAb were included in the medium to a final concentration of 5 μg/ml for 20 min, prior to co-incubation with target cells. In the experiments where MHC class I was blocked, the specific antibody (HP-1F7) was included in the medium at a final concentration of 10 μg/ml for 30 min. K562 cells were used as positive control targets.

### Measurement of IFNγ production

Natural killer cells were co-cultured with target cells for 6 h at 37°C 5% CO_2_ at an E:T ratio of 1:2. After 1 h of co-incubation, monensin was added to a final concentration of 2.5 μM. After 6 h, cells were recovered, fixed with 2% p-formaldehyde at RT for 10 min, permeabilized with 0.2% saponin at RT for 10 min, and intracellular IFNγ was analyzed by flow cytometry.

### Cytotoxicity assays

Bladder cancer cells were plated in triplicates at 1.5 × 10^5^ cells/ml, in a final volume of 1 ml, and labeled for 16 h with calcein AM (Molecular Probes, C3100MP) at 0.5 μg/ml. Cells were washed and incubated in fresh medium for 2 h. NK cells were co-cultured with target cells for 4 h at 37°C 5% CO_2_, at the indicated E:T ratios. Assays were terminated by collection of the supernatant from the co-culture, centrifugation at 200 × *g* for 5 min to eliminate remaining cells and collection of the new supernatant. Calcein release was determined by using EnVision^®^ Multilabel Reader, with these measurement parameters: excitation wave 486 nm and emission wave 530 nm. Specific lysis was expressed as percentage, calculated as the ratio [(value − spontaneous release)/(maximum − spontaneous release)] × 100. Spontaneous release was determined by incubation of the labeled target cells alone. Maximum release was determined by lysing the target cells in 0.1% SDS. In all the experiments, the spontaneous release was <25% of maximum release. Each experiment was repeated two to five times. Error was <5% of the mean of the triplicates. In blocking experiments, mAb was included to a final concentration of 5 μg/ml. K562 cells were used as positive control targets.

### Patient samples

Blood was obtained from a cohort of bladder cancer patients (Ta/T1 G3 or CIS, mean age 72.8) receiving BCG instillations, at different times during treatment at Hospital Infanta Sofía (Madrid, Spain). As a control, bladder cancer patients (Ta/T1 G2, mean age 70.1) receiving mitomycin C (MMC) instillations were recruited. The experiments were conducted with the understanding and the consent of each participant and approved by local and regional ethical committees (CEIC La Paz Hospital, CEI Infanta Sofía Hospital, and CSIC Local Ethical Committee). One hundred microliters of blood were analyzed by flow cytometry for the expression of CD56, NKG2D, and NKp46 using a Gallios Flow cytometer and Kaluza software. Data processing was performed creating an algorithm to compile information for further statistical analysis using the GraphPad Prism 6 package. Statistical significance was assessed using multiple *T*-tests and corrected for multiple comparisons using the Sidak–Bonferroni method.

## Results

### Bladder cancer cells can express different combinations of immune ligands

**Figure 1:**
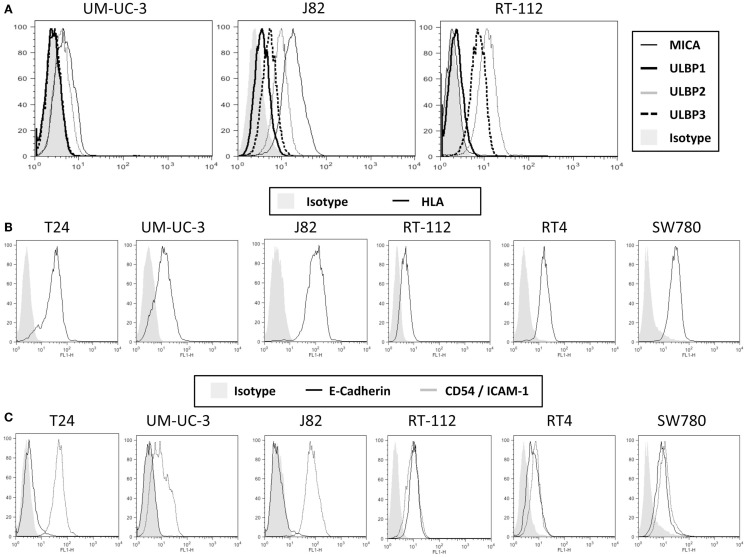
**Expression of ligands for NK cells on bladder cancer cell lines**. The indicated bladder cell lines were stained with antibodies specific for several NKG2D ligands **(A)**, surface MHC, using the pan-MHC antibody HP1F7 **(B)** and the adhesion molecules E-cadherin and CD54 **(C)**, and analyzed by flow cytometry. The figure shows a representative experiment out of more than five for each molecule. For more markers and statistical analysis, see Table 1 and Figure S1 in Supplementary Material.

To characterize the ability of bladder cancer cells to stimulate NK cell responses, a panel of urothelial tumor cells with different grades of differentiation (T24, UM-UC-3, J82, RT-112, RT4, SW780) were stained with antibodies against different NK ligands for flow cytometry analysis. Three groups of molecules were checked: ligands for activating receptors, inhibitory ligands, and adhesion molecules involved in NK recognition. Figure 1 shows representative flow cytometry profiles and Figure S1 in Supplementary Material the statistical analysis of all the markers studied. For simplicity, FACS data and grade of tumor cell lines are summarized in Table 1. Notably, different urothelial cells show different patterns of immune ligands as well as MHC and adhesion molecules, suggesting that their susceptibility to NK cell lysis could be different.

**Table 1:** **Cell surface expression of activating and inhibitory ligands by bladder cancer cells**.

		T24^b^	UM-UC-3^c^	J82^d^	RT-112^e^	RT4^e^	SW780^f^
	
	Tumor grade^a^	3	3	3	2	1	1
Activating and inhibitory ligands for NK receptors	MICA	+	+	+++	−	+	++
	ULBP1	−	−	−	−	−	−
	ULBP2	++	+	++	++	+	++
	ULBP3	++	+	+	++	++	++
	NKp46L	++	+	+	++	−	+
	NKp30L	+	+	+	+	+	+
	E-CADHERIN	−	−	−	++	++	++
	CD48	−	−	−	−	−	−
	MHC	++	+	+++	−	+	++
	HLA C	++	+	+++	−	+++	+
Adhesion molecules	CD54/ICAM-1	++	+	+++	+	+	+
	CD112/NECTIN	++	+	++	+	+	++
	CD155	++	++	++	+++	++	+++
	CD58/LFA3	++	++	++	+++	++	++
	CD106/VCAM-1	−	−	−	−	−	−

*Data summarize MFI values shown in Figure S1 in Supplementary Material*.

*^a^Source of tumor grade information: see reference beside each cell line name*.

*^b^Bubenik et al. (18)*.

*^c^Grossman et al. (19)*.

*^d^O’Toole et al. (20)*.

*^e^Masters et al. (21)*.

*^f^Kyriazis et al. (22)*.

All the cell lines analyzed expressed different levels of NKG2D ligands, all of which could contribute to the activation of NK cells. While all the cell lines showed low to medium levels of surface ULBP2 and ULBP3 (except UM-UC-3, that only had low levels of ULBP3), none of them expressed ULBP1; MICA surface levels show very different pattern among the different cell lines and MICB expression was not significant in any of the cell lines. To check for the capacity of each cell line for binding the NKG2D receptor, NKG2D-Fc constructs were used to stain the bladder cell lines (Figure S2 in Supplementary Material). Other activating ligands were also present at low amounts in urothelial cancer cells, like NKp30-ligand and variable amounts of NKp46-ligand (Figure S3 in Supplementary Material). The activating receptor 2B4 is present in most NK cells; however, its ligand CD48 was not detected in any of the bladder cell lines analyzed.

MHC plays an important role in NK cell inhibition, thus, the levels of total surface MHC and, in particular, HLA-C was also analyzed. Again, different levels were found on the different cell lines, J82 expressing highest amounts of MHC-I while the rest of the cell lines had lower levels. The amount of MHC observed at the cell surface of UM-UC-3 and RT-112 was very low or absent.

Although E-cadherin is an adhesion molecule, playing a pivotal role in Ca^2+^-dependent cell-cell adhesion and contributing to tissue organization and development, it has been shown to bind the killer cell lectin-like receptor G1 (KLRG1) that belongs to the C-type lectin-like superfamily and contains an ITIM in its cytoplasmic domain (23). T24, UM-UC-3, and J82 did not express surface E-cadherin, suggesting that this inhibitory interaction will not occur, while less dedifferentiated cell lines expressed this molecule. However, around 50% of IL-2-cultured NK cells did not express this receptor (data not shown), consistent with published data (24, 25).

Other surface molecules that could contribute to NK cytotoxicity were also analyzed. CD54 (ICAM-1, intercellular adhesion molecule-1) binds β_2_ integrins (such as LFA-1) and is crucial for the formation of conjugates (26, 27). All the bladder tumor cell lines analyzed expressed at least low amounts of ICAM-1 at the surface, suggesting that they all have what has been suggested as the minimal requirement for adhesion. However, different levels of expression could modulate the response. All bladder cells also expressed different amounts of CD112, CD155, and CD58. CD112 (Nectin-2, PVRL2) and CD155 (Necl5, PVR) bind CD226 (DNAM-1), which potentiates *in vitro* the cytotoxicity of NK cells against a wide range of tumor cells (28, 29); CD58 (LFA-3) binds CD2 and its co-engagement with ICAM-1 increases NK response (30).

None of the cell lines analyzed expressed surface CD106 (VCAM, vascular cell adhesion molecule), which strongly binds α_4_β_1_ integrins on lymphocytes (31).

In light of these data, different bladder cancer cell lines have several potential ligands to activate and inhibit NK cells and, since a large number of signals will need to be integrated it is difficult to predict the ability of each cell line to stimulate NK cell activation.

### Recognition of different bladder cell lines by NK cells depends mainly on NKG2D

**Figure 2:**
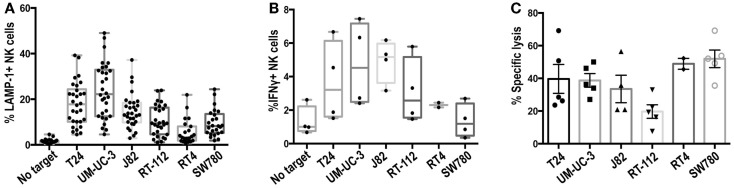
**Recognition of bladder cancer cells by IL-2-activated NK cells**. **(A)** Degranulation experiments. NK cells were incubated for 2 h with the indicated bladder cancer cells, pre-incubated with anti-MHC-I antibody, at an E:T ratio of 1:2 and surface LAMP-1 (CD107a) was analyzed by flow cytometry. **(B)** IFNγ production. NK cells were incubated with bladder cancer cells, blocked for MHC-I, for 6 h in the presence of monensin (2.5 μM) at an E:T ratio of 1:2 and IFNγ production was analyzed by flow cytometry. **(C)** Cytotoxicity experiments. Target cells were labeled with calcein for 16 h, washed and incubated for 4 h with NK cells at an E:T ratio of 10:1. Specific calcein release was measured in supernatants and the percentage of cytotoxicity calculated. Symbols represent results obtained with NK cells obtained from different donors.

Preliminary experiments showed that freshly isolated NK cells responded poorly to bladder cancer cells (data not shown). Thus, primary IL-2-activated NK cell lines were prepared from healthy donors and used as effector cells in degranulation experiments against the panel of bladder cancer cells. Figure 2A shows the percentage of NK cells that expressed LAMP-1 (CD107a) at the plasma membrane after contact with the target cell, a marker present in cytotoxic granules that allows identification of those cells that have activated the degranulation machinery. As expected from the variety of activating and inhibitory ligands expressed at the surface of different bladder cancer cell lines, NK cells responded differently to the panel of cell lines. Experiments were performed with NK cells prepared from a large number of donors to evaluate donor to donor variation. T24, UM-UC-3, and J82 were consistently better able to stimulate NK degranulation than RT-112, RT4, and SW780 with all the donors tested. The cell lines that stimulated the highest amount of degranulation were also most efficient in triggering IFNγ production (Figure 2B). However, all the bladder tumor cell lines, except RT-112, were killed efficiently in cytotoxicity assays (Figure 2C). This may reflect the involvement of non-lytic granule-dependent mechanisms of cytotoxicity, e.g., FAS or TRAIL-mediated death in causing the death of the RT-112, RT4, and SW780 cell lines.

**Figure 3:**
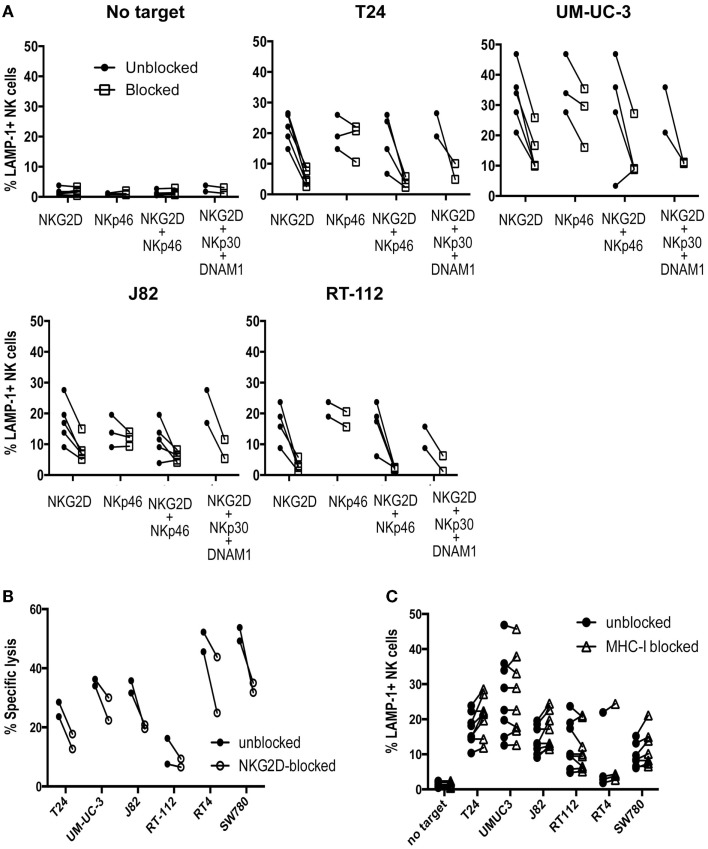
**Receptors involved in the recognition of bladder cancer cells by IL-2-activated NK cells**. **(A)** The effect of activating receptor blockade was studied in degranulation experiments measuring surface LAMP-1 (CD107a) by flow cytometry. NK cells were pre-incubated with antibodies against the indicated receptors before the co-incubation with bladder cancer targets for 2 h at an E:T ratio of 1:2. **(B)** The effect of NKG2D blockade was also analyzed in cytotoxicity experiments measuring specific calcein release from bladder target cells. Target cells were labeled with calcein for 16 h, washed and incubated for 4 h with NK cells at an E:T ratio of 10:1. Specific calcein release was measured in supernatants and the percentage of cytotoxicity calculated. **(C)** The effect of MHC blockade was studied in degranulation experiments measuring surface LAMP-1 by flow cytometry. Each panel shows values of several independent experiments for each cell line.

Although the cytotoxicity results could be explained in part by the presence of NKG2D ligands at the cell surface, other molecules probably play also a role. In order to identify NK receptors involved in the recognition of urothelial cells by primary activated NK cell lines, antibody blocking was performed in degranulation experiments. As shown in Figure 3A, NKG2D clearly contributed strongly to the recognition of bladder cancer cells while NKp46 had an additive role only in some of these cells probably due to the balance of signals. By contrast, addition of NKp30 or DNAM-1 blocking antibodies did not further enhance NKG2D blocking. Cytotoxicity experiments confirmed the importance of NKG2D for the recognition of bladder cancer cells (Figure 3B).

The contribution of inhibition mediated by recognition of MHC was also tested in experiments blocking MHC-I (Figure 3C). Although the cells expressing higher levels of MHC-I were recognized somewhat more efficiently after blockade, the loss of MHC inhibitory signals did not change the pattern of degranulation toward the different bladder cancer cells. Thus, these data confirmed that the variability on the lysis susceptibility among different bladder cell lines was independent of donor, and suggested that the lack of degranulation toward several of the cell lines did not depend on missing-self recognition.

A possible inhibitory effect of E-cadherin expression on NK cell recognition could be excluded since mAb-mediated blockade of KLRG1 had very little effect on NK degranulation (data not shown).

### Exposure of bladder cells to BCG did not affect susceptibility to NK lysis

**Figure 4:**
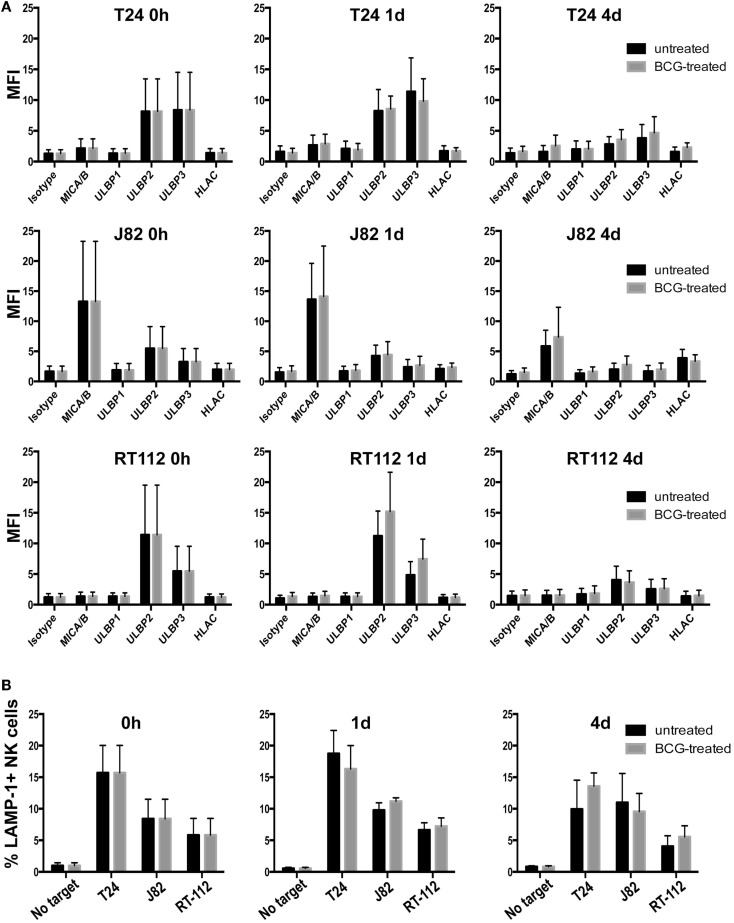
**Effect of BCG exposure on bladder cancer cell lines**. **(A)** Bladder cancer cell lines T24, RT-112, J82 were incubated in the presence of BCG (two bacteria per bladder cell) for the indicated times and analyzed by flow cytometry for the expression of NKG2D-L and MHC. Data show the average and SD of three independent experiments. **(B)** Degranulation experiments. Bladder cancer cell lines T24, RT-112, J82 were incubated in the presence of BCG for the indicated times and blocked with anti-MHC-I antibody before being co-incubated with NK cells for 2 h at an E:T ratio of 1:2. Data show the average and SD of three independent experiments.

After intravesical instillation of BCG in bladder cancer patients, mycobacteria first contact with the bladder epithelium and some reports indicate that urothelium could contribute to immune modulation. For example, urothelial cells have been shown to release cytokines after contact with *Escherichia coli* Hu734 (32) and to express toll-like receptors (33). In other experiments, exposure to *Mycobacterium tuberculosis* (Mtb) increased surface expression of one of the NKG2D-L (34). For these reasons, we investigated whether exposure to BCG affected the presence of NKG2D ligands on the surface of a panel of bladder cancer cell lines. Cells were initially treated with BCG for 1 and 4 days and no major changes were observed in the expression of NKG2D ligands (Figure 4A), then longer times were analyzed and, up to day 7 no differences were observed (data not shown). Since it is difficult to estimate how many bacteria encounters each bladder cell during treatment, the experiments were performed using several ratios of BCG:bladder cells (1:1–10:1) and no difference in surface NKG2D ligands was observed. Similar data were obtained with two other strains of BCG. To investigate if BCG-exposed bladder cells were also recognized by IL-2-activated NK cells through other receptor–ligand interaction, degranulation assays were performed after treating urothelial cells with BCG (Figure 4B). Surprisingly, only minor changes were observed in the recognition of these cells.

### Exposure of IL-2-activated NK cells to BCG did not affect recognition of bladder cancer cells

**Figure 5:**
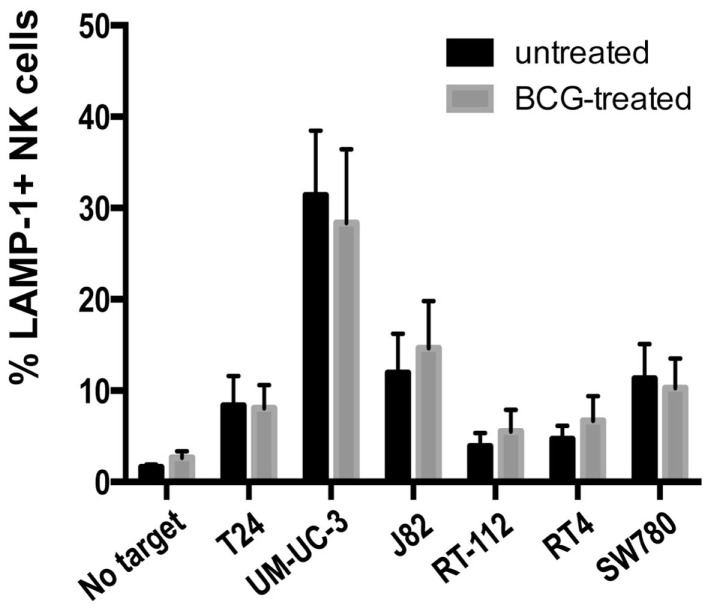
**Effect of BCG exposure on IL-2-activated NK cells**. The ability of BCG-exposed NK cells to respond to bladder cancer cells was analyzed in degranulation experiments measuring surface LAMP-1 (CD107a) by flow cytometry. NK cells were incubated with BCG (1:1) for 4 days and used as effectors in degranulation assays with the indicated bladder cancer cells blocked with anti-MHC-I antibody. Data show the average and SD of three independent experiments.

Previous reports showed that activation of PBMCs with BCG lead to recognition of the urothelial cell line T24 (6, 7, 9). Next, we studied whether IL-2-stimulated NK cells could change their ability to recognize a panel of bladder cancer cells after exposure to BCG for four days. Unexpectedly, no substantial changes were observed (Figure 5), indicating that, once IL-2 activated, NK cells from healthy donors respond to bladder cancer cells independently of the presence of BCG. Similarly, BCG treatment of freshly isolated, purified NK cells had no effect on degranulation in response to bladder cell lines (data not shown). These data suggest that the importance of BCG for immunotherapy resides in the events that lead to NK cell recruitment and activation, probably in the context of other subpopulations of immune cells.

### NK cell activation in the presence of PBMCs and BCG achieves a better response to bladder cancer cells

**Figure 6:**
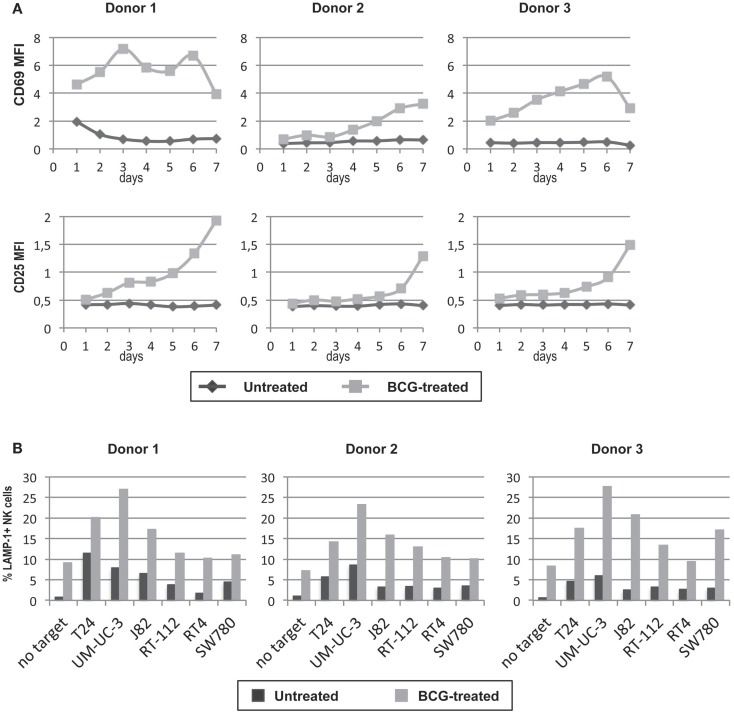
**Effect of BCG exposure on NK cells in culture with other lymphocytes**. NK cells were incubated with BCG (1:1) for 7 days and their activation phenotype was analyzed by flow cytometry analyzing surface CD25 and CD69 at various time points during the culture **(A)**. PBMCs were used as effectors in degranulation assays with the indicated bladder cancer cells and the response by NK cells was evaluated gating in the CD3^−^CD56^+^ region. The ability of NK cells previously cultured with BCG to respond to bladder cancer cells was analyzed in degranulation experiments measuring surface LAMP-1 (CD107a) on the CD3^−^CD56^+^ population of PBMC **(B)**. Data show three experiments with different donors.

In view of the previous experiments, where exposure to BCG neither affected the recognition of bladder cell lines by IL-2-activated NK cells nor led to activation of purified NK cells. We next studied the phenotype and function of NK cells in cultures of PBMCs cultured for 7 days either in the presence or absence of BCG. After 1 week in culture, only the NK cells that had been in presence of BCG acquired an “activated” phenotype (as judged by expression of CD69 and the IL-2-receptor α-chain CD25), while NK cells in the control culture remained “resting” (Figure 6A). In addition, BCG-activated NK cells degranulated better than unstimulated NK cells against the panel of bladder cancer cells (Figure 6B). Interestingly, BCG-activated NK cells improved their degranulation against all the bladder cancer cells, indicating that this activation process is more efficient than just IL-2 activation. These data indicate that crosstalk between immune cells stimulated by the presence of BCG leads to an activation of NK cells that improves their anti-tumor response.

### The percentage of peripheral blood NK cells varies among BCG-treated bladder cancer patients but not with treatment

**Figure 7:**
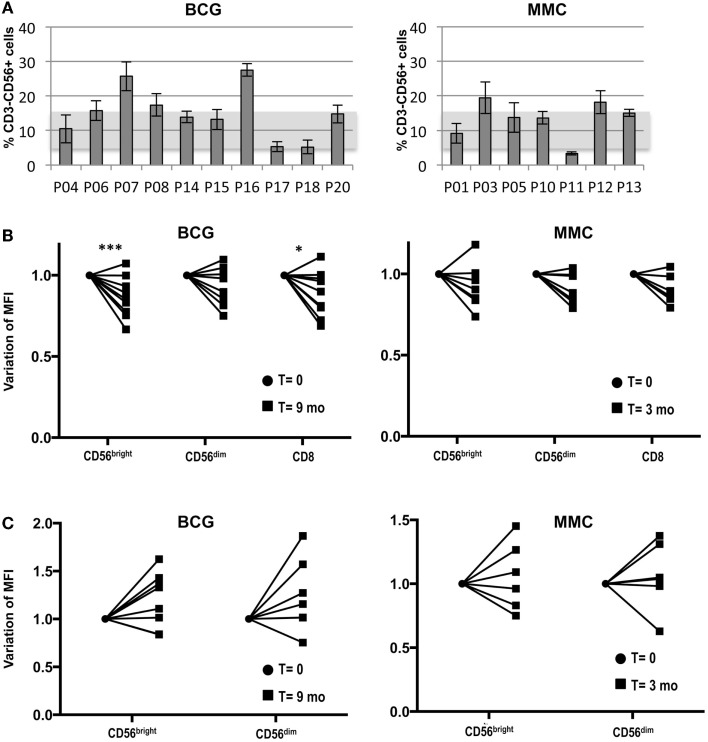
**Study of the NK cell compartment in a cohort of bladder cancer patients treated with BCG**. Blood samples were collected from bladder cancer patients at different time points during treatment and analyzed by flow cytometry. **(A)** Percentage of NK cells. The graph depicts the average and SD of the percentage of CD3^−^CD56^+^ cells in lymphocytes for each patient during the first 9 months of treatment (nine samples for each patient). Usual values in healthy donors are represented by a shadow. **(B)** NKG2D. The mean fluorescence intensity of NKG2D was determined in different lymphocyte populations of the patients at different times (12 time points for BCG-treated patients; 4 time points for MMC-treated patients). NKG2D MFI ranged between 30 and 80 in CD56^bright^, 30 and 70 for CD56^dim^, and 40 and 70 in CD8^+^ T cells, within this patient cohort (data not shown). The value of the NKG2D MFI from the initial (●) (before starting treatment) and final samples (■) (9 months for BCG; 3 months for MMC) were normalized for comparison and statistical analysis. The significance of the variation between the initial and final sample was analyzed by *t*-test using the Sidak–Bonferroni method. *Significant (*p* < 0.05) ***significant (*p* < 0.01). **(C)** NKp46. The MFI of NKp46 was determined in the different lymphocyte populations, normalized and analyzed by *t*-test using the Sidak–Bonferroni method as in B. NKp46 MFI ranged between 2 and 18 in CD56^bright^ and 1.5 and 10 for CD56^dim^, within this patient cohort (data not shown). The increase in NKp46 MFI was not significant.

Given that healthy activated NK cells respond to bladder cancer cells via NKG2D, we next analyzed the expression of this receptor in the NK cell compartment in peripheral blood of BCG-treated cancer patients. The percentage of NK cells in peripheral blood of a cohort of BCG-treated bladder cancer patients (*N* = 10, T0–T1 G3) was evaluated as part of a larger pilot study underway in the laboratory. The expression of the NK marker CD56 was analyzed by flow cytometry at different time points of the treatment for each patient. Figure 7A shows the average of the NK cell percentage for each patient during the first 9 months of treatment. The SD of these data reflect that the number of circulating NK cells did not vary much for each patient with the treatment, instead, it was very different from donor to donor. For comparison, bladder cancer patients (*N* = 7, T1G2) receiving intravesical instillations of MMC were included and the distribution of NK cells in peripheral blood of these control patients was similar to that of the BCG-treated patients.

The levels of NKG2D on peripheral blood cells were also analyzed (Figure 7B). Although NKG2D expression by CD4^+^ T cells has been reported in some diseases (35, 36), it was not observed in these patients (data not shown). As expected, all CD8^+^ T cells and NK cells were positive for NKG2D. However, different patterns and values could be observed in the different patients. In general, NKG2D was brighter in CD56^bright^ cells, compared to CD56^dim^ cells (data not shown). Interestingly, while the variation during treatment in the levels of NKG2D in PBMCs of MMC-treated patients was not significant, the surface expression of NKG2D by CD56^bright^ and CD8^+^ cells of the BCG-treated patients did decrease significantly over the course of the treatment. Interestingly, while NKG2D expression decreased in the majority of patients, a few patients did not follow this trend. NKp46 was expressed mainly in CD56^bright^ cells and, in most BCG-treated patients, an increase that did not reach statistical significance in the variation over the time of therapy of the expression of this receptor was observed (Figure 7C).

## Discussion

Natural killer cells had been suggested to play a role in the recognition of J82 and T24 bladder tumor cell lines (6, 7, 9). However, no systematic analysis of receptors and ligands involved in the interaction between NK cells and a large panel of bladder tumor cells has been reported. So, the first objective of this work was to investigate the mechanism of recognition of bladder tumor cells by IL-2-activated NK cells and the role of several NK receptors mediating degranulation and cytoxicity toward these targets. We describe that different bladder cancer cells express, at their cell surface, a large number of ligands that can contribute to the activation and inhibition of effector cells. In aggregate, all the bladder cell lines studied express different NKG2D ligands and the contribution of the NKG2D receptor was dominant. When the degranulation experiments were compared with the phenotype of bladder cancer cell lines, it could seem initially that surface expression of NKG2D-L would not explain the differences in NK cell activation. For example, UM-UC-3 and T24, both strong stimulators for NK cell degranulation, expressed less NKG2D-L than J82. However, the large amount of NKG2D-L on J82 might be counteracted partially by the presence of other inhibitory ligands, among them, MHC-I. Interestingly, E-cadherin was found only in those cell lines corresponding to lower grade tumors, while high-grade cell lines did not have this molecule at the cell surface. This could imply that E-cadherin could play an important role as a negative modulator for the outcome of the interaction with low-grade tumor cells. The functional consequences of the interaction between KLRG1 and cadherins are still under study, but it is clear that the receptor can modulate the immune response of both NK and T cells (37, 38). However, blockade of KLRG1 on IL-2-activated NK cells did not change the degranulation observed (data not shown), ruling out the possibility that this interaction would be important in this system. When the data from antibody blocking experiments were analyzed, it was interesting to note that, although blocking the NKp46 by itself does not have a major effect on degranulation, the addition of NKp46 blocking mAb to NKG2D blockade often blocks NK recognition of the tumor more strongly. This synergistic effect of NKp46 is consistent with previous reports (39). In this context, similar to the panel of bladder tumor cell lines studied here, primary non-muscle invasive bladder tumors have also been reported to express ligands for all three natural cytotoxicity receptors (40). Yet, in our functional assays, only NKp46 blocking mAbs had any effect on NK cell recognition. Similarly, although the RT-112 and SW780 cell lines expressed more CD155 than the other cell lines, blocking DNAM-1 with antibody did not have any clear effect on degranulation suggesting that, at least in our system, the possible interaction of CD155 with either DNAM-1 or CD96 (TACTILE), a receptor expressed on resting NK cells that competes with CD226 and could be a negative regulator (41), is not a critical influence on NK recognition.

Thus, in summary, the main receptor involved in the recognition of bladder cancer cells by NK cells is NKG2D, which is present in all NK cells. However, in some cases, modulation of the response by NKp46 and adhesion molecules can contribute to immune recognition by NK cells in this system. NKG2D-specific mAb blocked recognition of bladder cancer cells both in degranulation and cytotoxicity experiments, however it is interesting to note that some cell lines that did not trigger much degranulation by the NK cells, were clearly dying in cytotoxicity experiments. This could be related to death independent from lytic granules, such as Fas–FasL, TNFα, or TRAIL-mediated interactions. In fact, it has been reported that a bladder cell line, T24, but not J82, is susceptible to FasL-mediated lysis (4).

One of the major therapeutic strategies for bladder cancer is the generation of an inflammatory/immune response to the tumor by intravesical instillation of BCG. The mechanism of action of this therapy is not fully understood, but several lines of evidence suggest that NK cells play a key role in this response (4–9). Thus, a second objective of the work presented here was to test whether exposure of the bladder tumor cell lines to BCG affected NK cell recognition. Our results show that BCG exposure on bladder cancer cells affected neither NK cell degranulation nor the expression of NKG2D ligands by these cells. NKG2D-L are upregulated in cells exposed to Mtb (34, 42), pointing to a difference between the two bacteria species. The data further show that treating either IL-2-activated NK cells or freshly isolated NK cells with BCG did not affect the intensity of the response against several bladder cancer cell lines. Interestingly, however, stimulation of resting NK cells with BCG in PBMC cultures did improve their anti-tumor response and revealed that their activation status is consistent with cytokine secretion. In particular, the upregulation of CD25 indicates that BCG might be stimulating the secretion of IL-2 presumably by T cells within the culture. These data suggest that the particular manner to trigger NK cell activation might be a key factor in the treatment of bladder cancer with BCG, and that the participation of other blood subpopulations is necessary to achieve the appropriate NK response. More experiments are required to understand in detail the process of NK cell activation in the context of an ongoing immune response against BCG.

Finally, the observation of significant changes in NKG2D expression by NK cells in the blood of patients undergoing BCG therapy supports the conclusion of the *in vitro* experiments that NKG2D is a key receptor for immune recognition in bladder cancer. Moreover, since the number of NK cells in patient peripheral blood did not vary during the treatment of bladder cancer with BCG it will be important to extend these observations to study NK cell activation both locally and in peripheral blood. It has been established that NKG2D is downmodulated after interaction with its ligands (43), so the decrease in the receptor could reflect that an interaction with cellular or soluble ligands has occurred. These patient data also indicate that in-depth study of the phenotype of these effector cells to evaluate the response may lead to the identification of possible biomarkers of the NK cell activation in each patient.

In conclusion, here we demonstrate that NK cells can recognize bladder tumor cells, mainly through NKG2D, and that BCG exposure facilitates this anti-tumor response by modifying the activation status of NK cells, not by modifying the receptors directly involved in the cytotoxic response.

## Conflict of Interest Statement

The authors declare that the research was conducted in the absence of any commercial or financial relationships that could be construed as a potential conflict of interest.

## Supplementary Material

The Supplementary Material for this article can be found online at http://journal.frontiersin.org/article/10.3389/fimmu.2015.00284/abstract
