# Supplementary figures and images for: NKG2D is a Key Receptor for Recognition of Bladder Cancer Cells by IL-2-Activated NK Cells and BCG Promotes NK Cell Activation
Source: Front Immunol. 2015 Jun 8;6:284. doi: 10.3389/fimmu.2015.00284 (PMC4459093; doi:10.3389/fimmu.2015.00284)

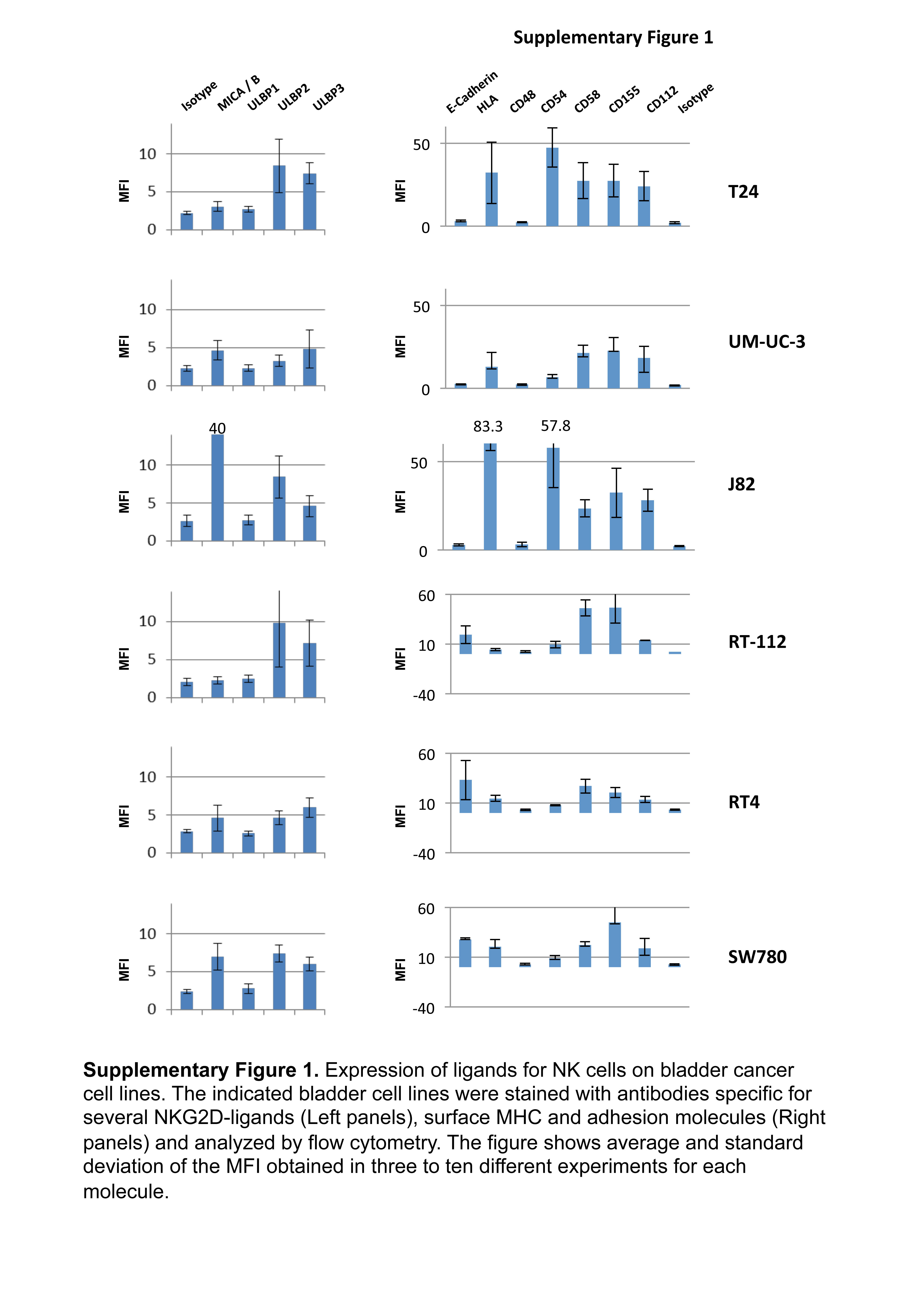

Supplement: Supplementary file 1 [file Image_1.JPEG]

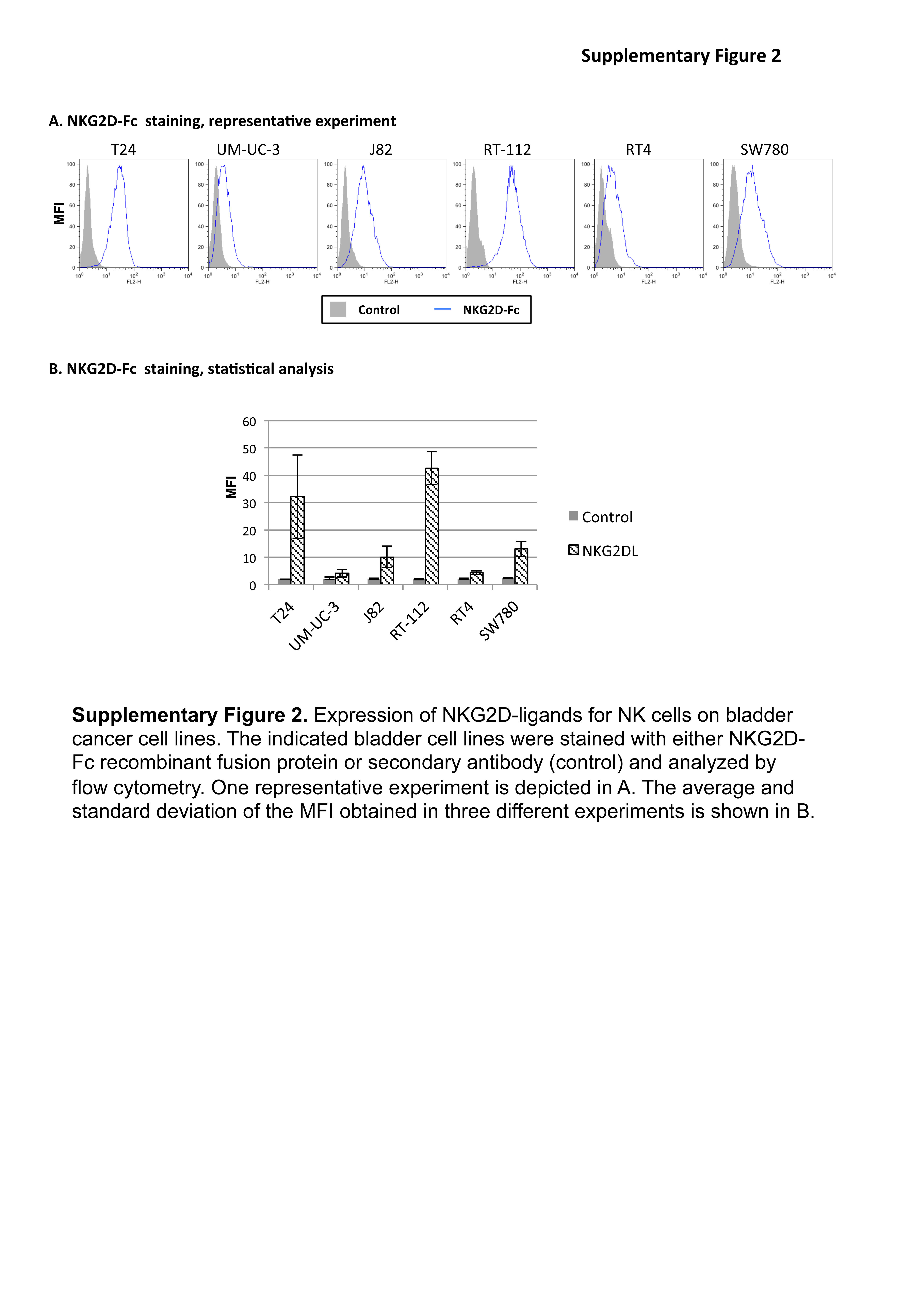

Supplement: Supplementary file 2 [file Image_2.JPEG]

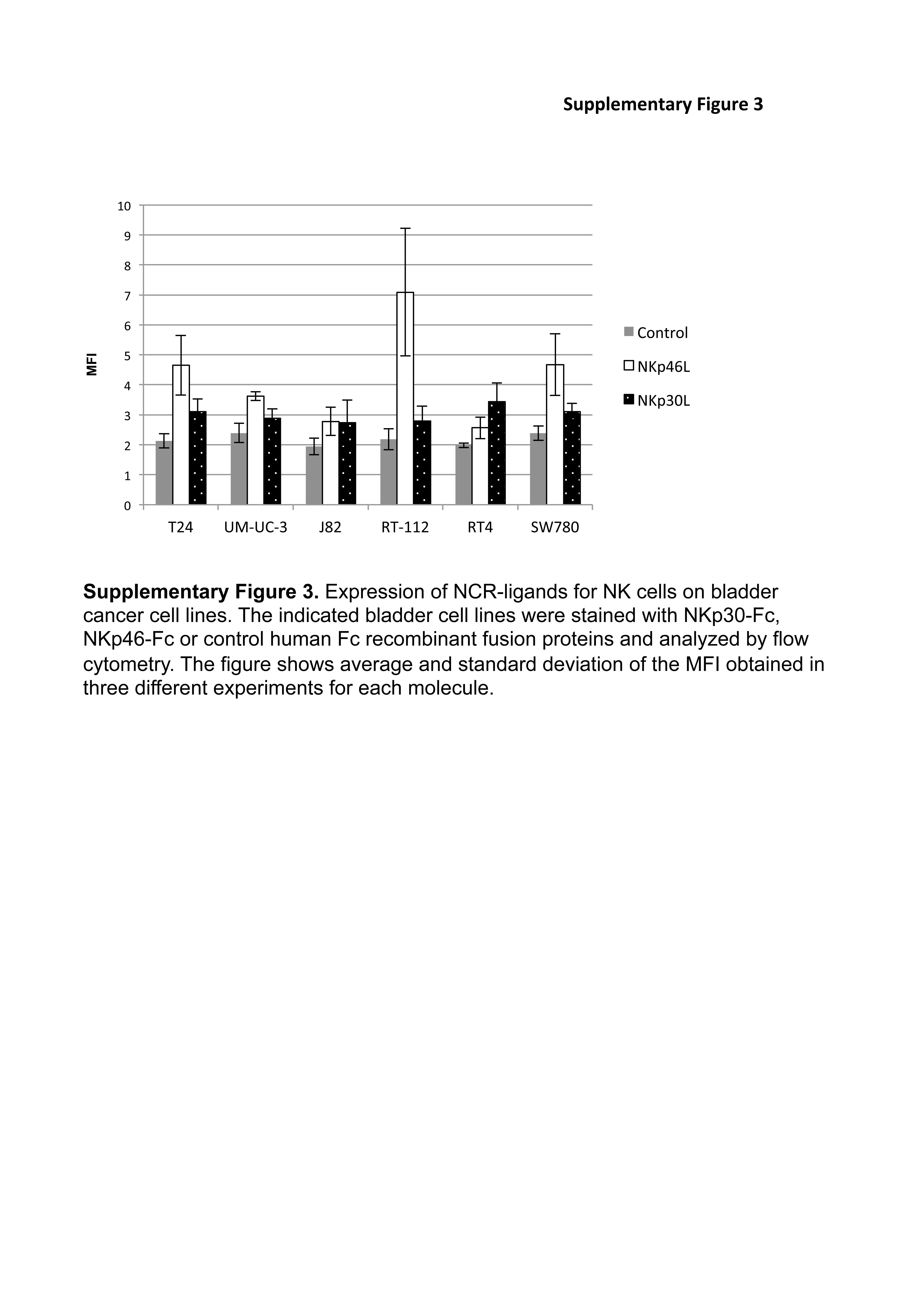

Supplement: Supplementary file 3 [file Image_3.JPEG]
